# Leaf Extracts of *Anchomanes difformis* Ameliorated Kidney and Pancreatic Damage in Type 2 Diabetes

**DOI:** 10.3390/plants10020300

**Published:** 2021-02-05

**Authors:** Toyin Dorcas Alabi, Nicole L. Brooks, Oluwafemi O Oguntibeju

**Affiliations:** 1 Phytomedicine & Phytochemistry Group, Oxidative Stress Research Centre, Department of Biomedical Sciences, Faculty of Health and Wellness Sciences, Cape Peninsula University of Technology, Bellville 7535, South Africa; toyulabs@gmail.com; 2 Faculty of Health and Wellness Sciences, Cape Peninsula University of Technology, Bellville 7535, South Africa; brooksn@cput.ac.za

**Keywords:** *Anchomanes difformis*, apoptosis, diabetes, inflammation, aqueous extract, nephropathy, oxidative-stress

## Abstract

Kidney disease in diabetes is one of the common microvascular complications of diabetes mellitus implicated in end-stage renal failure. This study explored the ability of *Anchomanes difformis* to ameliorate kidney and pancreatic damage in type 2 diabetes mellitus using male Wistar rats. Two weeks of fructose (10%) administration followed by streptozotocin (40 mg/kg) were used to induce type 2 diabetes. Leaf extract (aqueous) of *Anchomanes difformis* (200 mg and 400 mg/kgBW) was administered orally for six weeks. Body weights were monitored, urea and creatinine were measured. Interleukins (IL)-1β, IL-6, IL-10, IL-18, and TNFα were measured in the kidney lysate. CAT, SOD, ORAC, FRAP, and MDA levels were also evaluated in the kidney. Transcription factors (Nrf2 and NF-ĸB/p65) and apoptotic markers (Bcl2 and caspase 3) were investigated in the kidney. Histological sections of the pancreas and kidney tissues were examined for any visible pathology. Supplementation with *Anchomanes* *difformis* enhanced antioxidant status, modulated inflammatory response, and reduced apoptosis in the kidney. It also restored the kidney and pancreatic histoarchitecture of the treated diabetic rats. The pathophysiology associated with diabetic nephropathy and pancreatic damage showcase the importance of exploring the use of antidiabetic, nephroprotective agents such as *Anchomanes difformis* to kidney damage in type 2 diabetes.

## 1. Introduction

Kidney disease in diabetes (referred to as diabetic nephropathy—DN) is a common complication of type 2 diabetes mellitus (T2DM) and has become one of the major causes of kidney failure [1,2]. It is widely characterised by abnormalities in the architecture of the renal tissues, such as expansion of the mesangial matrix, glomerular hypertrophy, thickening of the glomerular, and tubular basement membrane [3]. Abnormal values of urea, creatinine, albumin and other proteins in the serum and urine are typical observations in diabetic subjects [2]. Administration of fructose and low dose of STZ causes partial disruption of the beta cells leading to less production of insulin and hence, hyperglycemia with insulin resistance [4,5]. Hyperglycemia leads to oxidative stress by influencing the generation and overproduction of ROS through the increased activation of the polyol pathway, protein kinase C (PKC) and formation of advanced glycation end-products (AGEs) [1,6]. An imbalance in the production of ROS and the antioxidant (such as catalase and superoxide dismutase) results in oxidative stress [7,8]. Oxidative stress induced by hyperglycemia can further inhibit the production of insulin by the beta cells of the pancreas. This occurs through hyperglycemia-mediated activation of uncoupling protein-2 leading to leakage of protons and reduced ATP/ADP ratio in the beta cells. ROS produced as a result of constant hyperglycemia can also occasion lipid peroxidation in the cell membrane resulting in beta cell damage [1,9]. The excessive production of ROS such as superoxide anion in pancreatic beta cells can activate stress-signalling pathways, which further induces inflammatory and apoptotic transcription factors, such as NF-ĸB, this leads to beta cell death and reduced insulin production [9].

NF-ĸB is upregulated during oxidative stress in most cells of the kidney including tubular cells, mesangial and endothelial cells and the podocytes [10]. The activated NF-ĸB causes the transcription of proinflammatory genes coding for cytokines (TNFα, IL-1β, IL-2, IL-6, IL-12, and IL-18) and chemokines (MCP-1). NF-ĸB also enhances the transcription of profibrotic genes responsible for growth factors (TGF-β) and leukocyte adhesion molecules (E-selectin, VCAM1, and ICAM-1). The expression of these proinflammatory and profibrotic proteins triggers inflammation, atherosclerosis and vascular dysfunction [11].

Cell death is a key factor in the progression of DN [12]. Chronic exposure of tissues to oxidative stress sufficiently induces a wide range of pathophysiological events that leads to eventual cell death [3]. In normal tissues, unwanted cells are removed by apoptosis thereby maintaining tissue homestasis, however, in damaged cells, apoptosis is triggered thereby activating cell-death receptors such as TNFRs [12,13,14]. As a result of the inappropriate activation of these intracellular signalling pathways, an imbalance occurs between cell death and cell proliferation in the damaged tissues in diabetes [15,16]. The expression of Bcl2, an anti-apoptotic protein, is majorly detected in the proximal and distal tubules and in the capsular parietal cells in normal rats and humans [17]. Interestingly, a loss of balance has been recorded in DN between pro- and anti-apoptotic markers, such as the decreased expression of anti-apoptotic Bcl2 protein [18]. Furthermore, increased expression of Growth factors such as TGF-B are induced by hyperglycemia and oxidative stress in the glomerulus and other tissues. The upregulation of TGF-B promotes further, the generation of ROS by activating NADPH oxidase and mitochondrial respiratory process resulting to aggravated TGF-B-induced apoptosis and detachment of the podocyte [19,20].

**Figure 1:**
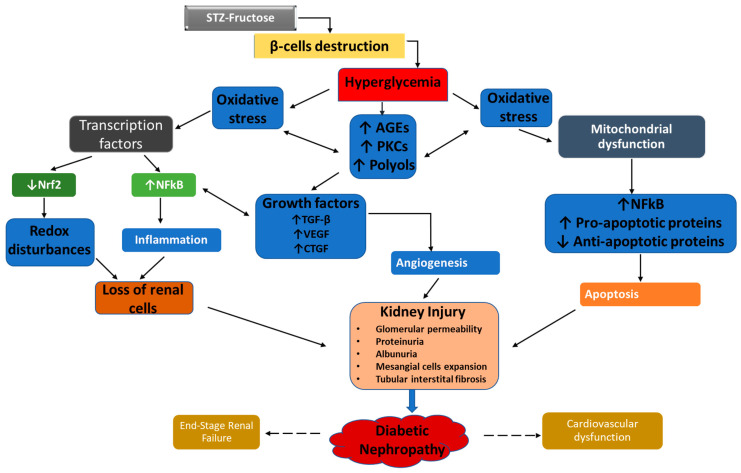
Pathogenesis of diabetic nephropathy.

ROS-mediated renal injury finally progresses into chronic kidney disease by evoking hemodynamic dysregulation and abnormalities in the structure and function of the nephron [21]. The damaged glomerulus and its filtration barrier increase permeability of plasma proteins such as albumin (albuminuria), a crucial process involved in the etiology of DN [3]. Elevated proteins (in the tubular ultrafiltrate) in the presence of increased ROS elicit various aberrant signaling pathways [3]. The increased activation of these impaired signalling mediators which includes transcription factors, inflammatory agents, growth factors, and vasoactive molecules causes several pathological events in the glomerulus and the tubules promoting renal injury from a progressive state to an end-stage renal failure [3]. Figure 1 summarizes the key pathways and mediators involved in the progression and pathogenesis of DN.

There is a paradigm shift towards the use of medicinal plants in the management of pathological conditions and diseases, due to its cost effectiveness, with little or no adverse effects when appropriately used. It is also more available when compared with conventional drugs [22,23]. *Anchomanes difformis* (AD) of the family. Araceae has been used traditionally against varying pathological conditions, including diabetes, kidney pains, asthma, pain and wounds, microbial infections, and gastrointestinal related problems. Some of these folkloric uses and claims have been proven with scientific studies, while others are still indigenous claims [24]. We carried out a study on the identification and characterization of the bioactive compounds present in six different extracts of AD using HPLC and UPLC-MS, antioxidant capacities of these extracts were investigated. Aqueous extract exhibited the highest antioxidants ability [25]. The results also revealed that AD extracts especially aqueous, contains phytochemicals such as apigenin, phloridzin, kaempferol, rutin, catechin, chlorogenic acids amongst others which are active against hyperglycaemia, oxidative stress, inflammation, and apoptosis [25,26]. The wide range of biological properties of compounds present in AD has necessitated the need to further investigates its potentials against diabetes and diabetic complications. Our research works then investigated, established and reported the antidiabetic ability of AD aqueous extract [27], which led to further explore the attenuating ability of AD diabetic complications involving the liver, heart and the reproductive system [27,28,29]. However, no study has been conducted to investigate the potency of AD leaves on the kidney and pancreas pathology in diabetes mellitus. This study therefore investigates the ameliorative potential of AD in hyperglycemia-induced kidney and pancreatic damage in diabetic male Wistar rats.

## 2. Results

### 2.1. Effect of Treatment with AD on the Relative Weight of the Kidney and Pancreas

**Figure 2:**
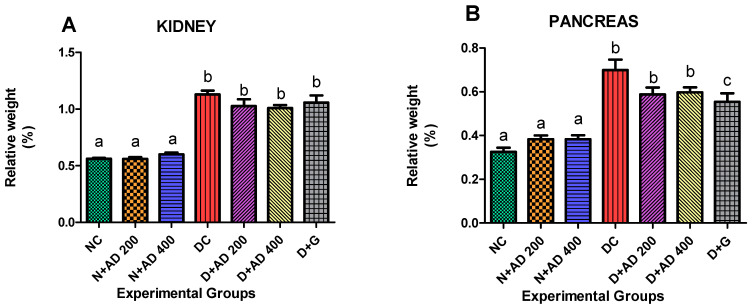
Effect of AD administration on the (**A**) relative kidney weight and (**B**) relative pancreas weight of normal and diabetic rats. Bars are indicative of mean values ± SEM of group values. Bars with different letters are significantly (*p* < 0.05) different from each other.

Figure 2 shows the relative weight of the kidney and pancreas in normal and diabetic rats. Induction of diabetes using fructose and STZ led to a significant increase in the relative weights of the kidney and pancreas in the untreated diabetic rats (positive control). However, intervention with 200 mg and 400 mg/KgBW AD lowered relative kidney weight by 9.1% and 10.7% respectively (Figure 2A). Glibenclamide (5 mg/KgBW) also reduced relative kidney weight by 6.4% in the treated diabetic rats. Similarly, relative pancreatic weight was significantly increased in the positive control when compared with the normal rats (Figure 2B). The relative pancreatic weight was minimized in the treated rats by 15.9% and 14.6% following the administration of 200 mg and 400 mgKgBW AD. This is comparable to the activity of glibenclamide which had a 20% reduction on the relative pancreatic weight (Figure 2B).

### 2.2. Effect of AD Administration on Kidney Function Markers

**Figure 3:**
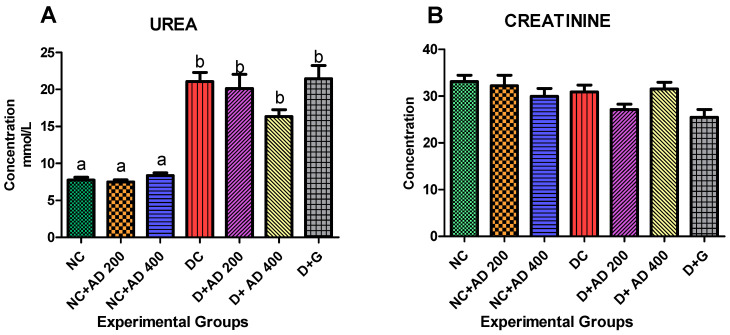
Effect of AD administration on the (**A**) urea and (**B**) creatinine concentration in the serum of normal and diabetic rats. Bars are indicative of mean values ± SEM group values. Bars with different letters are significantly (*p* < 0.05) different from each other.

Hyperglycemia caused by STZ and fructose administration resulted in significant increase (by 3 folds) in the serum levels of urea in the diabetic rats. Treatment with 400 mg/kgBW and 200 mg AD abated the urea concentration by 22.5% and 4.5% in treated diabetic rats when compared with untreated diabetic rats (Figure 3A). Administration of 5 mg/kgBW glibenclamide did not reduce urea concentration in diabetic kidneys when compared to the untreated diabetic kidneys. Creatinine levels in the serum was not significantly affected by either hyperglycemia or treatment with AD (Figure 3B).

### 2.3. AD Enhanced the Antioxidant Status in the Kidney

**Figure 4:**
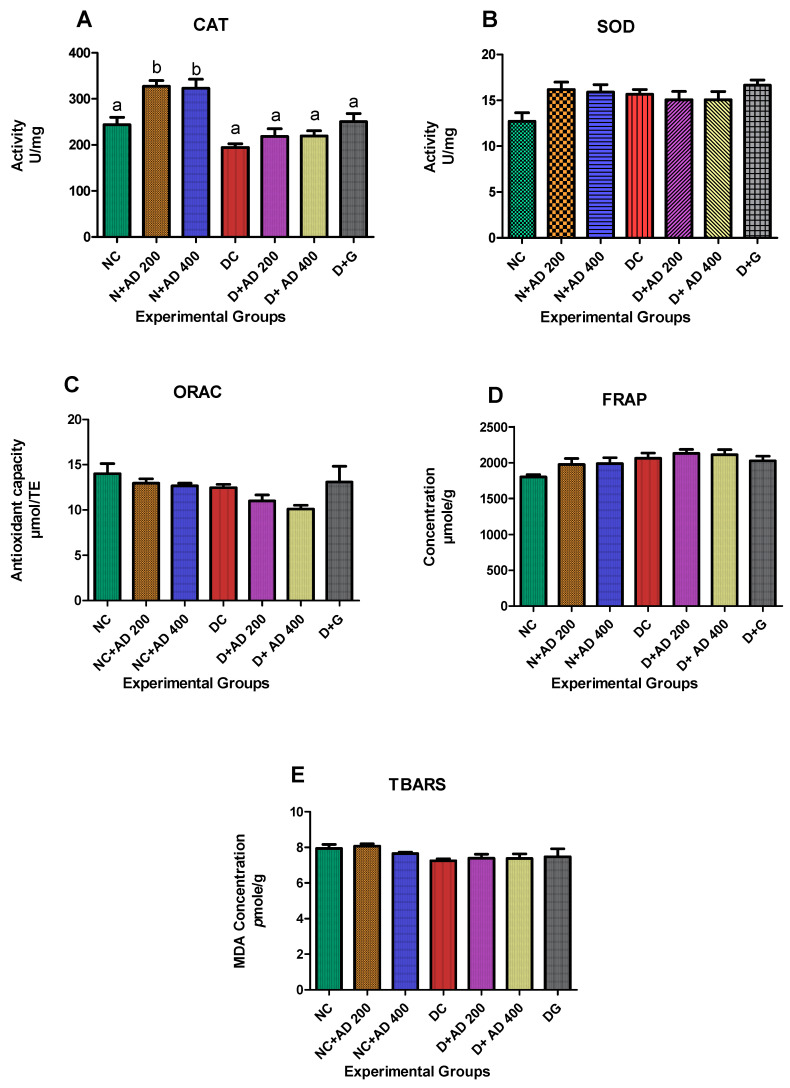
Effect of intervention with AD on the antioxidant capacities; (**A**) CAT, (**B**) SOD, (**C**) ORAC (**D**) FRAP and (**E**) Lipid peroxidation in the kidney of normal and diabetic rats (TBARS). Bars are indicative of mean values ± SEM of group values. Bars with different letters are significantly (*p* < 0.05) different from each other.

The effect of AD and glibenclamide on the antioxidant indices (CAT, SOD, ORAC and FRAP) and lipid peroxidation marker (TBARS) is presented in Figure 4A–E. The administration of AD significantly increased CAT levels in the normal rats treated with 200 and 400 mg of AD (Figure 4A). Conversely, a significant decrease (20.2%) in the activity of CAT was observed in the diabetic control when compared with the normal rats. However, supplementation with 200 mg and 400 mg/KgBW of AD led to about 13% increase in the activity of CAT in the treated groups when compared with untreated diabetic rats (Figure 4A). In addition, the activity of SOD was increased by 27% and 25% in the normal rats treated with 200 mg and 400 mg/KgBW respectively when compared with the normal control. There was no significant difference in the ORAC, FRAP, and TBARS levels in the kidney of diabetic control, treated, and normal rats.

### 2.4. AD Modulated Hyperglycaemia-Induced Immune Response in the Kidney

**Figure 5:**
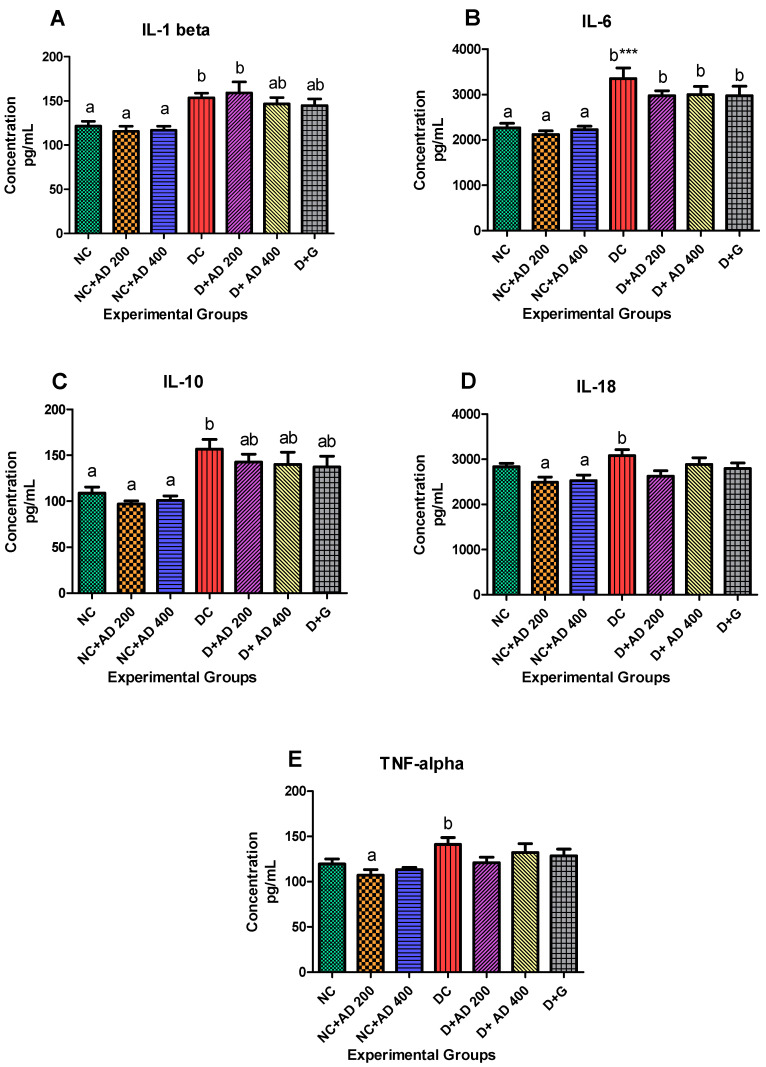
Effect of AD administration on interleukins (IL) (**A**) IL-1β, (**B**) IL-6, (**C**) IL-10 (**D**) IL-18 and (**E**) TNF-alpha in the kidney of normal and diabetic rats. Bars are indicative of mean values ± SEM of group values. Bars with different letters are significantly (*p* < 0.05) different from each other, while bars with *** is significantly different at *p* < 0.0001.

Induction of diabetic condition triggered increased inflammatory response in the diabetic rats as IL-1β and IL-6 were significantly elevated in untreated diabetic rats (Figure 5A,B). Intervention with 400 mg AD and 5 mg glibenclamide showed a significant reduction in the IL-1β levels in the treated diabetic kidney. An appreciable decrease (11%) was observed in the kidney levels of IL-6 of rats treated with both concentrations of AD and glibenclamide, when compared with untreated diabetic rats. There was an 8.6% increase in the kidney levels of IL-18 in the untreated diabetic rats (Figure 5D). However, 200 mg and 400 mg/KgBW of AD significantly abated IL-18 in the kidney of treated diabetic rats by 14.7% and 6.3% respectively when compared with the positive control. It is noteworthy that supplementation with 200 mg and 400 mg/KgBW AD decreased IL-18 levels by 12.2% and 10.9% in the normal treated kidneys when compared with the normal control rats (Figure 5D). This same trend was observed in the TNFα levels in the treated normal kidneys (Figure 5E). 200 mg and 400 mg/KgBW AD curtailed the expression TNFα by 10% and 5% respectively in the non-diabetic treated rats. Diabetes triggered TNFα response (15%) in untreated diabetic kidney. Then, 200 mg and 400 mg/KgBW AD supplementation reduced TNFα by 14.4% and 6.3%, respectively, in treated diabetic rats when compared with diabetic control (Figure 5E). IL-10 levels were significantly increased in the kidney of diabetic control rats, this was modulated in diabetic rats placed on AD extract, when compared to the diabetic control (Figure 5C). A similar trend was observed in the diabetic rats treated with glibenclamide.

### 2.5. Effect of AD Supplementation on Transcription Factors in Normal and Diabetic Rats

**Figure 6:**
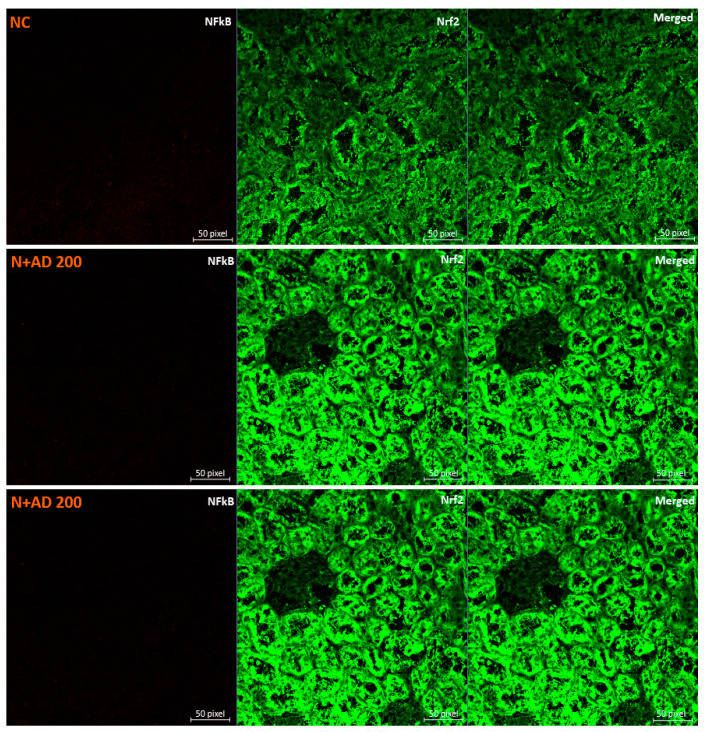

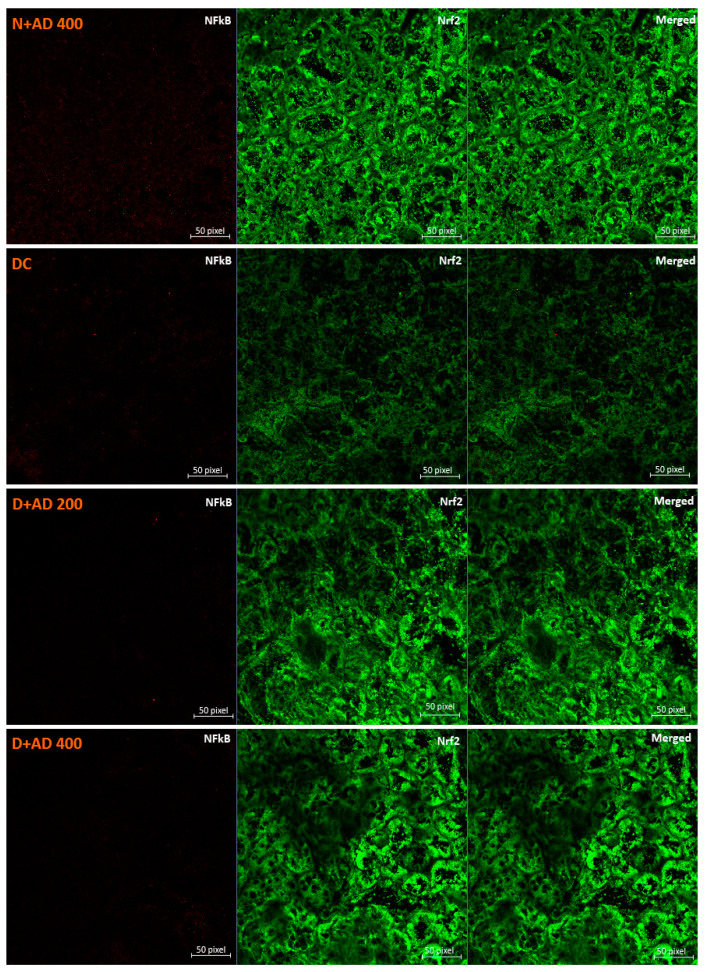

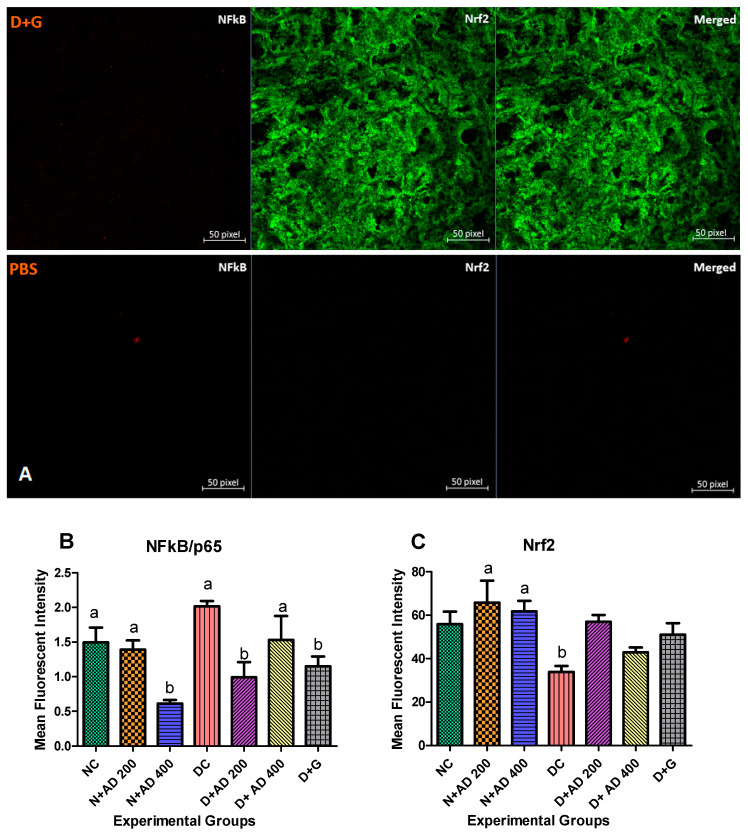
(**A**) Confocal microscopy image showing the effect of AD on the expression of NF-ĸB/p65 (red) and Nrf2 (green) in the kidney tissues. Quantitative analysis of (**B**) NF-ĸB/p65 and (**C**) Nrf2 expression in the Kidney tissues. Bars are indicative of mean values ± SEM of group values. Bars with different letters are significantly (*p* < 0.05) different from each other.

NF-ĸB expressions in the kidney of untreated diabetic rats were upregulated as depicted by the 35% increase in the MFI when compared with the normal control rats (Figure 6B). Management with 200 mg and 400 mg/KgBW of AD brought about 50% and 25% reduction in the NF-ĸB/p65 levels in the treated diabetic kidney. Treatment with glibenclamide also led to 43% reduction in the expressions of NF-ĸB/p65. This was comparable to the levels observed in the normal rats (Figure 6B). The expressions of Nrf2 were significantly enhanced in the treated normal rats placed on 200 mg and 400 mgKgBW of AD (Figure 6A). This was significantly dwindled (40%) in the untreated diabetic rats. Intervention with 200 mg and 400 mg/KgBW AD elevated Nrf2 expressions (40% and 27.3% respectively) in the treated diabetic kidney (Figure 6C). A similar upregulation (33%) was observed in the groups placed on 5 mg/KgBW glibenclamide.

### 2.6. AD Administration Upregulated the Expressions of Anti-Apoptotic Proteins in Diabetic Rats

**Figure 7:**
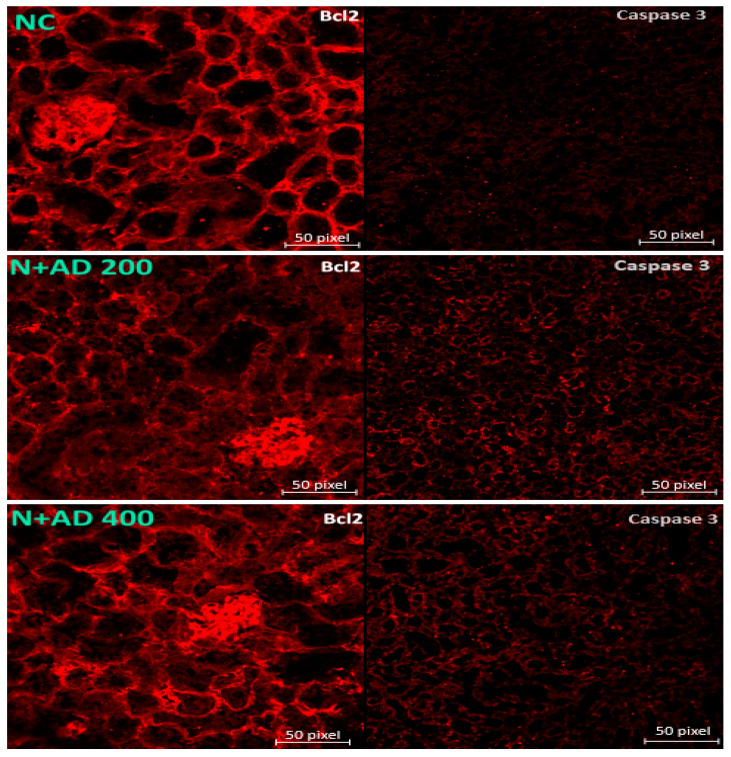

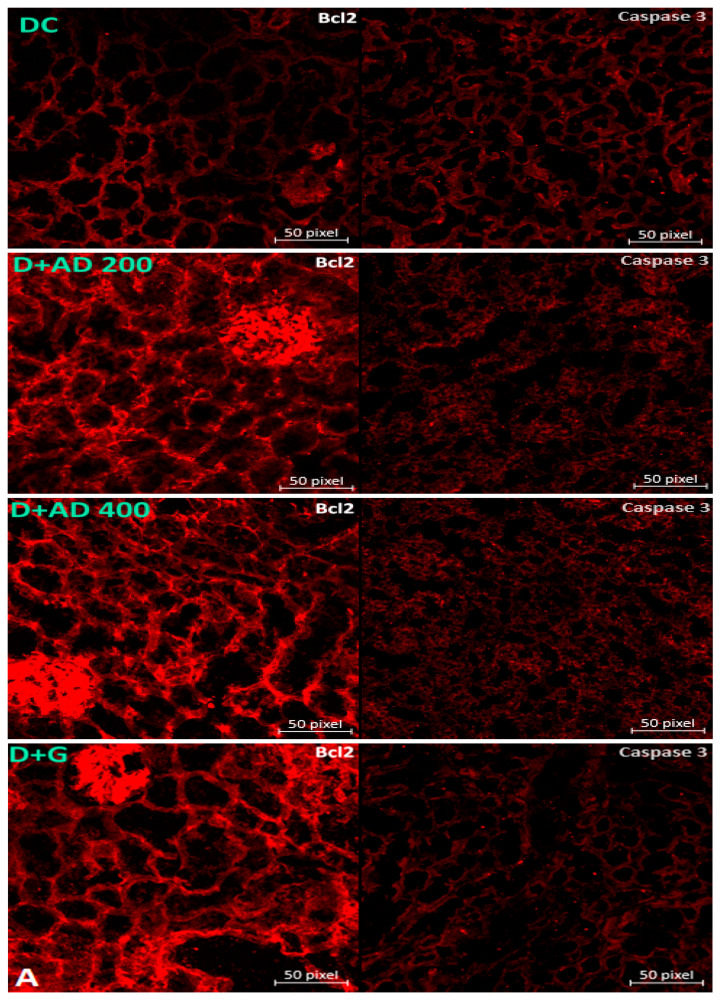

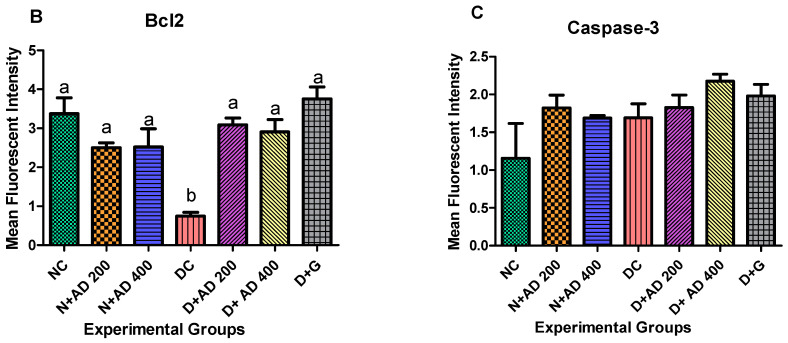
(**A**) Fluorescence micrographs showing the effect of AD intervention on apoptotic markers in the kidney tissues of normal and diabetic rats. Quantification of the level of expression of (**B**) caspase 3 and (**C**) Bcl2 in the kidney tissues.

The induction of diabetes altered Bcl2 expressions in the untreated diabetic kidneys. This led to a significant decline in the MFI of Bcl2 in the untreated diabetic rats when compared with normal control rats (Figure 7A). A regimen of 200 mg and 400 mg/KgBW of AD significantly restored the Bcl2 levels as comparable to the observed levels in the normal control rats (Figure 7B). STZ-induction of diabetes or treatment with AD did not have any significant difference in the expressions of caspase 3 (Figure 7A,C).

### 2.7. Histology

#### Intervention with AD Improved Histoarchitecture of the Kidney and Pancreas in Type2 Diabetes

**Figure 8:**
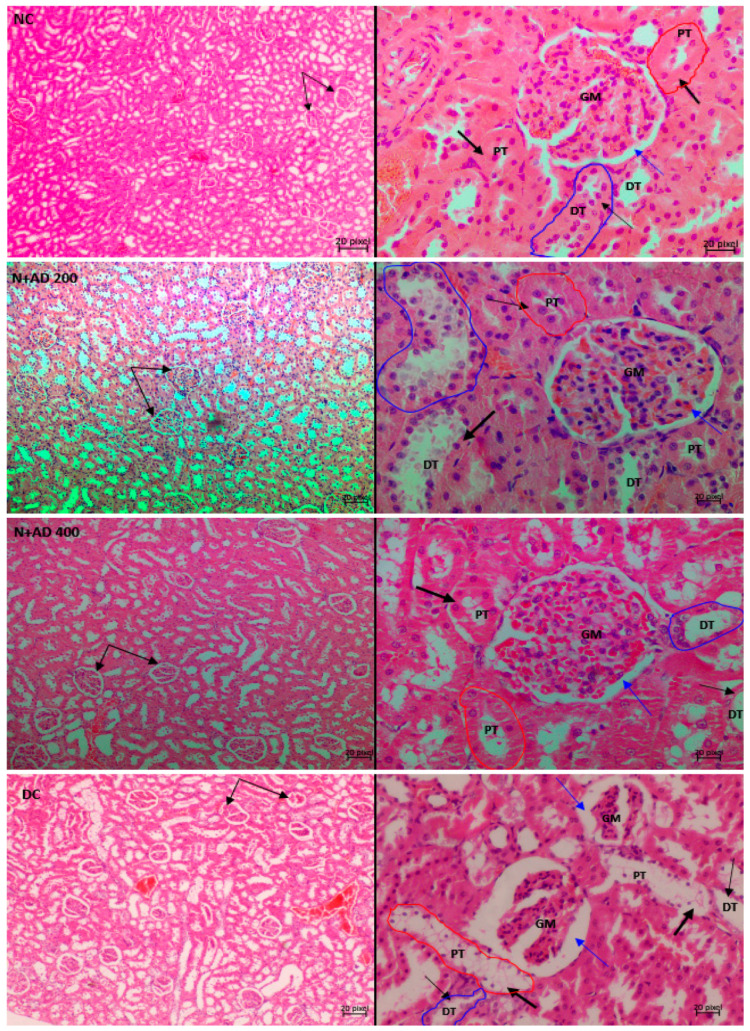

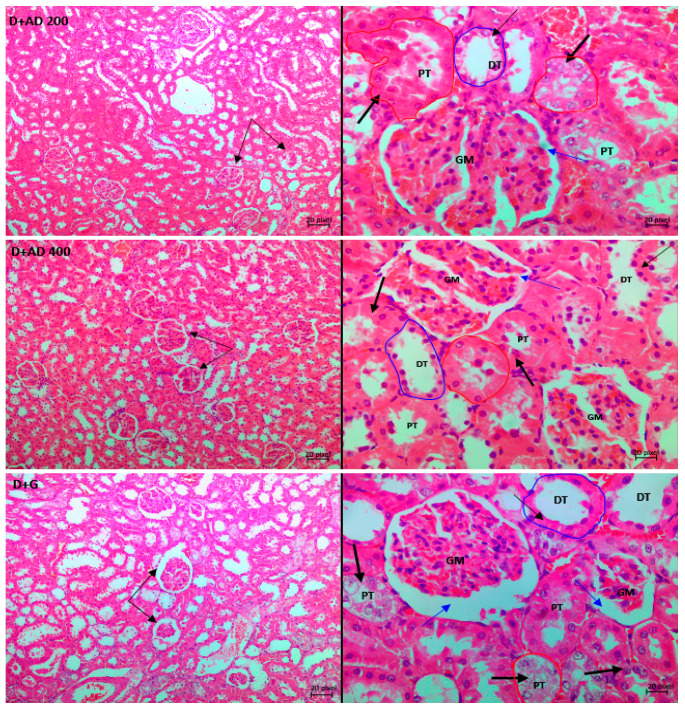
Light photomicrographs of haematoxylin and eosin-stained kidney cortex of normal and diabetic rats. Double arrows are pointing to the bowman’s capsule and glomerulus (GM), while single thin arrows are showing the cells of the distal convoluted tubule (DT). The thick single arrows reveal the cells of the proximal convoluted tubule (PT). The blue arrows points at the bowman’s space. Magnification 10× (**left**) and 40× (**right**).

**Figure 9:**
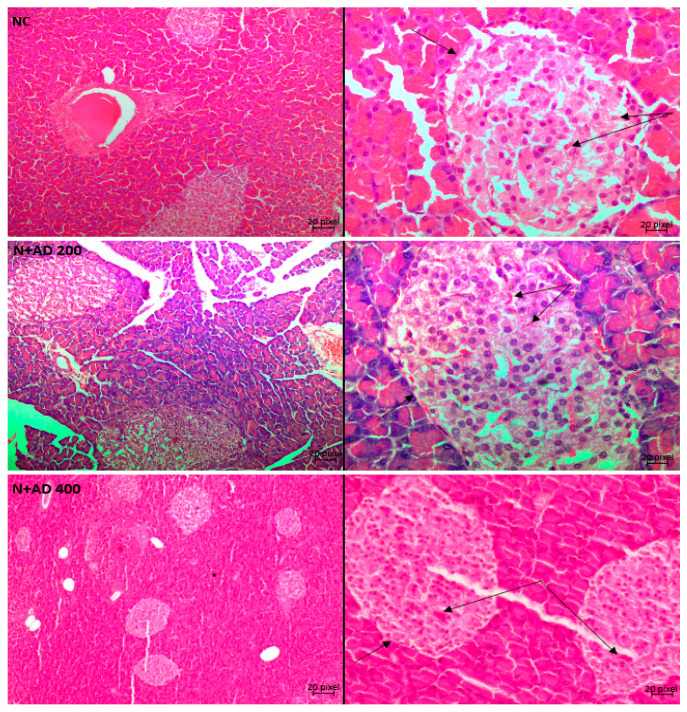

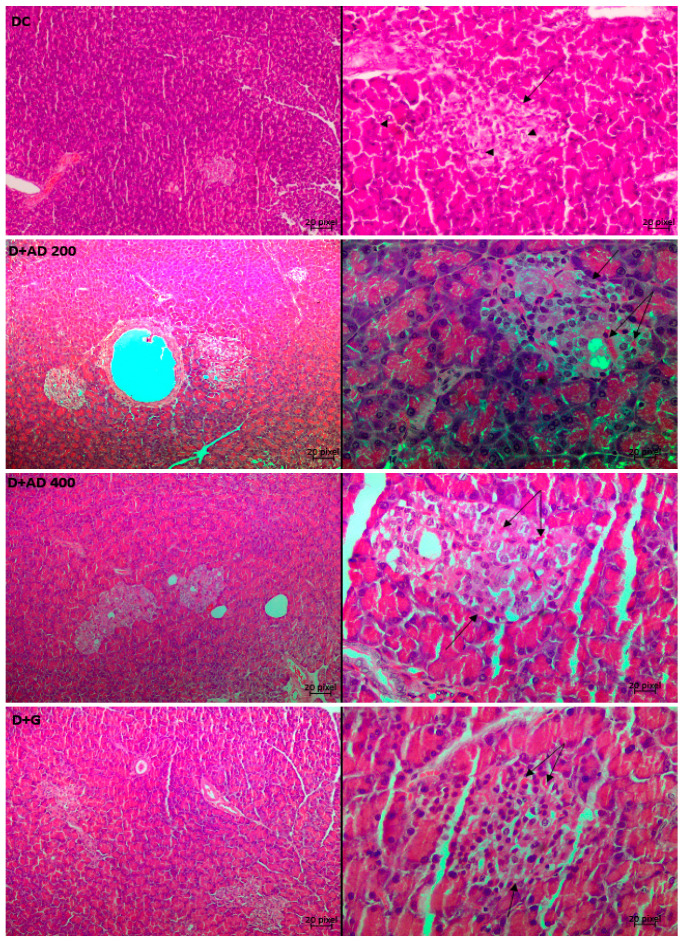
Light photomicrographs of haematoxylin and eosin-stained pancreatic tissue of normal and diabetic rats. Single arrows point to the basement membrane of the islets, while double thin arrows are showing the islet capillaries. Arrow heads show vacuolated nuclei in the acinar and islet cells. Magnification 10× (**left**) and 40× (**right**).

Figure 8 and Figure 9 are representative micrographs of the histological examination in the kidney and pancreas tissues of the normal, treated and untreated diabetic rats. Normal control and treated normal rats showed normal kidney architecture. Pathological conditions such as disappearing of the glomerular tuft comprising of the glomerular capillaries, podocytes and mesangial cells were observed in the kidney tissues of untreated diabetic rats. This led to widened Bowman’s space in the diabetic control rats when compared with the kidney of normal rats. Furthermore, endothelial cells were markedly detached from the basement membrane of the glomerulus in the untreated diabetic kidneys. Loss of tubular cells especially of the proximal tubules (PT) and increased blood flux were revealed in the kidney sections of untreated diabetic rats. Treatment with 200 mg/KgBW showed mild loss of tubular cells in the PT, while glomerular capillaries were clearly restored and less occurrence of glomerular shrinkage. Then, 400 mg/KgBW AD ameliorated glomerular and tubular damage in the kidney of treated diabetic rats. 400 mg/KgBW prevented loss of tubular cells of the proximal tubules and minimized loss of glomerular tuft in the treated diabetic rats as comparable to normal. The administration of glibenclamide showed widened Bowman’s space due to shrinkage of the glomerular tuft and moderate loss of tubular cells of the PT.

The histological examination of the pancreatic tissue in the non-diabetic rats showed a normal histology of the pancreas (Figure 9). The exocrine region clearly distinct from the endocrine region as the basement membrane of the islets of Langerhans is visible and the islet appeared lightly stained. The shape and structure of the islets is not distorted, and the pancreatic lobules are intact, separated by interlobular and intralobular spaces. The islet was well vascularized (double arrows). STZ induction of diabetes revealed pathological changes in both the endocrine and exocrine regions of the pancreas in the positive control rats. The structure and shape of the islet of Langerhans were distorted as the basement membrane is not visible (single arrow), severe destruction of the islet cells especially the beta cells are also observed. Vacuolation of the nucleus is seen in almost all the acinar cells and the islet cells. Distortion of the berry-like shaped acini was displayed, the pyramidical shape of the acinar cells were also distorted. Treated diabetic rats placed on 200 mg/KgBW of AD showed a better structured acinus, acinar cells, and the islet with restoration of beta cells when compared to the untreated diabetic rats (Figure 9). However, pathological alterations such as moderate vacuolation of the acinar and islet cells were seen. Supplementation with 400 mg/KgBW of AD restored the architecture of the pancreas to normal when compared with the untreated diabetic rats. The islets were well vascularized, the structure and shape of the islet were normal, and the basement membrane is clearly visible. There was no observable damage such as vacuolation in the acinar and islet cells. Also, the structure and shape of the acinar cells were restored. Treatment with glibenclamide improved the histology of the pancreas but not comparable to normal. The shapes of the acini and its cells were moderately distorted, and the basement membrane of the islet not distinct enough.

## 3. Discussion

The current study exhibits the protective effect of AD against organ toxicity, immunotoxicity and oxidative stress exerted by STZ on the kidney and pancreas in T2D. Organ weights have been used as important markers in investigating the toxicity of xenobiotics [30] and are indicative of hypertrophy if significantly increased [31]. Relative weight changes of internal organs are indices of pathology in the organs [32]. Introduction of fructose and STZ brought about significant increase in the relative weight of the kidney and pancreas of diabetic rats. Renal toxicity, tubular hypertrophy is known to be reflective of changes in the kidney weight [33]. Also, constant hyperglycemia results in the formation of AGEs and hyperfiltration leading to glomerular hypertrophy [34,35]. Administration of AD apparently reduced kidney toxicity as revealed by its ability to decrease relative kidney weight by 10%; an improvement over treatment with glibenclamide, which had no effect. Similar trends were observed in the relative weight of the pancreas in the diabetic control which increased significantly when compared to normal rats. However, AD abated abnormally elevated pancreatic weights in treated diabetic rats comparable to glibenclamide. This depicts that AD may be useful in minimising or preventing kidney and pancreas toxicity.

Urea and creatinine are one of the major metabolic waste excreted in the urine [3]. Abnormal levels of urea in the serum is indicative of kidney dysfunction [36]. Serum urea levels was significantly increased in the diabetic controls when compared to normal rats. There were severe pathological changes seen in the glomerulus of the diabetic control such as disappearing glomerulus tuft and capillaries, widened bowman’s space which are essential in glomerular filtration. Abnormal glomerular filtration may lead to increased urea and creatinine in the serum. Also, tubular damage especially in the proximal tubules was observed in the kidney tissues of untreated diabetic rats. Proximal tubules are very crucial in the reabsorption process as most essential substances in the filtrate including urea are reabsorbed in the proximal tubule [3,37]. Therefore, the elevated levels of serum urea were likely due to the glomerular and tubular damage. Similar results were revealed by other studies [36,38] where serum urea was significantly raised in diabetic subjects. Conversely, the administration of AD, diminished serum concentration of urea in treated diabetic rats, especially the 400 mg/KgBW treatment which reduced serum urea by 22.5%. This is expected as 400 mg/KgBW of AD restored the architecture of the kidney as comparable to normal rats. It is noteworthy that AD was more effective in restoring organ function in the diabetic rats than glibenclamide, as glibenclamide did not have any reducing effect on the serum urea. Also, corresponding pathologies were seen in the tubules and glomeruli of rats placed on glibenclamide.

Flavonoids have been established to ameliorate diabetic kidney damage by combating oxidative stress, reducing serum urea, blood urea nitrogen, creatine and infiltration of inflammatory markers [39,40]. Phytochemical analysis on AD showed that it contains flavonoids including apigenin, catechin, phloridzin, rutin, and kaempferol which possess antioxidant, anti-inflammatory, anti-apoptotic and hypoglycemic properties [25,26] that are key players in the amelioration of diabetes and diabetic complications. The flavonoid content of AD suggestively culminates to its ability to attenuate diabetic-induced nephropathy.

**Figure 10:**
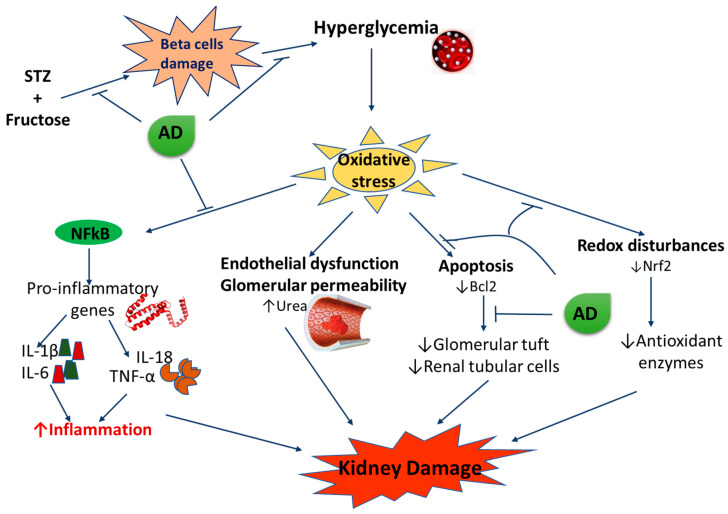
Proposed mechanisms by which AD ameliorates diabetic nephropathy and pancreatic damage. AD-*Anchomanes difformis.*

Oxidative stress triggered by persistent hyperglycemia plays an important role in the progression of diabetic nephropathy [35,41]. Oxidative stress occurs as a result of imbalance in the production of ROS and antioxidants. AD was shown to induce CAT and SOD activities in normal rats, thereby improving the antioxidant status in the rats. The significant decline in the CAT activity in the untreated diabetic kidneys is indicative of oxidative stress. In contrast, AD elevated CAT levels in the kidneys of treated diabetic rats. Increased activities of CAT and SOD by AD, suggestively reduced oxidative stress in the diabetic kidneys thereby ameliorating injury to cells and resultant cell death that may have occurred as revealed in Figure 10. Previous studies on the phytochemical content of AD confirmed the presence of antioxidant compounds [25]. A slight impaired induction of SOD levels was observed in the diabetic control which is likely as a response to the hyperglycaemic-induced oxidative stress. Lim and colleagues [42] reported a similar finding of impaired induction of SOD and a corresponding reduction in SOD activity in diabetic nephropathy.

Increased Nrf2 levels are known to be enhanced by oxidative stress, however, Nrf2 levels can be activated by in the presence of phytochemicals with antioxidant ability [43]. Supplementation with AD stimulated increased production of Nrf2 in normal and diabetic rats (Figure 6C). One of the possible mechanisms by which AD exerts its antioxidant ability may be that the antioxidant compounds present in AD trigger the dissociation of Nrf2/Keap1, activating Nrf2. Activated Nrf2 binds to the promoter region of the AREs gene, enhancing the transcription of AREs genes that code for antioxidant enzymes such as CAT and SOD. Figure 4A showed that there was a corresponding increase in the CAT and SOD activities of rats treated with AD, which follows the pattern of Nrf2 induction in the same rats. The significant decrease of Nrf2 observed in untreated diabetic rats concurs with the reduced CAT activities in the same rats. Benipal and co-researchers [44] hypothesized that repetitive ROS production downregulates the expression of Nrf2.

NF-ĸB activated by oxidative stress; plays a central role in inflammatory mediation and its deregulation is strongly associated with inflammatory diseases, hence its role in the progression of renal damage [45,46]. NF-ĸB expression was upregulated in the kidney of diabetic control rats, which was downregulated in the diabetic rats placed on AD. The activation of NF-ĸB usually promotes the expression of pro-inflammatory markers and apoptosis. The increased production of the inflammatory markers such as TNFα further activates NF-ĸB leading to prolonged and persistent inflammatory response [47,48].

Pro-inflammatory indices IL-1β and IL-6 were significantly increased in the kidneys of diabetic control rats when compared to normal rats. Proinflammatory cytokines IL-1β, IL-6, IL-18, and TNFα which play major roles in the development and progression of DN are triggered by hyperglycemia and oxidative stress [49]. Increased expression of IL-1β in the glomeruli of STZ-induced diabetes and significant increase of IL-6 in diabetic subjects especially in the mesangium, interstitium, and the tubules have been documented [50]. The study on kidney biopsies of DN subjects carried out by Navarro-Gonzalez and Mora-Fernandez [50], showed a positive correlation of IL-6 expression in the mesangium and podocytes with glomerular injuries. IL-18, a strong proinflammatory agent that influences the production of other proinflammatory cytokines (IL-1β and TNFα), is also elevated in DN, which in turn increases urinary albumin excretion thereby increasing kidney susceptibility to renal damage [51,52,53,54]. High levels of TNFα in DN patients are implicated in microalbuminuria [51], which promotes the generation of ROS locally in cells such as mesangial cells and activates transcription factors, growth factors, receptors and other cytokines [3,50] in the renal cells. TNFα can also alter the glomerular filtration rate, cause a dysfunction in endothelial permeability and eventual induction of apoptosis [3]. However, the administration of AD led to decrease in the levels of these markers, as comparable to the glibenclamide in the diabetic treated rats. Similar trends were observed in the levels of IL-18 and TNFα. Supplementation with AD is a potential therapy against inflammation-induced renal injury in T2D as also supported by its suppression of NF-ĸB and reduction of pro-inflammatory cytokines in the normal rats (Figure 5A–E and Figure 6A,B). The abnormal increase in the levels of IL-10 in the diabetic rats may be due to compensatory response, this was significantly repressed in the rats administered AD and glibenclamide.

Bcl2 acts as an anti-apoptotic protein, regulates apoptosis and promotes cell survival by inhibiting pro-apoptotic proteins [55]. Bcl2 also acts as an anti-inflammatory protein by inhibiting NF-ĸB and its controlled genes. The expression of Bcl2 was significantly reduced in the untreated diabetic rats. Studies have reported decreased Bcl2 expression in diabetic subjects [17,44]. This is expected as poor glycemic control represses the expression of Bcl2 in diabetes [55]. Reduced expression of Bcl2 leads to increased inflammation and apoptosis which contributes to the progression of renal pathology in diabetic subjects [55]. AD regimen significantly restored the expressions of Bcl2 to normal in treated diabetic rats. Lau and co-workers [56] investigated the underlying mechanisms through which apoptosis occur in DN. They found out that Bcl2-modifying factor (Bmf), a pro-apoptotic protein is induced by hyperglycemia via ROS and growth factor (TGFβ1) and upregulated in diabetic kidneys of rats and humans. Activated Bmf translocates from the cytoskeleton to the mitochondria where it binds and inhibits Bcl2 and other apoptotic proteins. This ultimately generates mitochondria transmembrane potential, which activates the intrinsic signalling pathway of apoptosis. It was also observed that Bmf can activate caspase 3 leading to apoptosis [55].

The major pathological alterations observed in the kidney tissues of untreated diabetic rats were disappearing glomerular tuft due to loss of podocytes, mesangial cells and glomerular capillaries. Also, loss of tubular cells especially the proximal tubules was observed in diabetic controls. This was ameliorated in the diabetic rats treated with AD. Apoptotic cell death plays a very crucial role in the loss of mesangial cells and podocytes [57,58]. Studies have reported the apoptotic death of renal tubular cells across various diabetic models such as mice, rats and humans [56]. One of the main causes of renal and glomerular apoptosis is increased inflammation. NF-ĸB, a pivotal inflammatory factor is activated in the podocytes, mesangial cells and tubular cells [10]. Increased expression and activation of NF-ĸB in these cells results in the production of pro-inflammatory factors and eventually apoptosis [47]. Decreased apoptosis was reported in NFκB tubular epithelial-specific knockout mice due to less activation of NFκB and reduced chemokine expression [48]. This supports the central role played by NF-ĸB in the apoptosis of the tubular and glomerular cells. A strong correlation has been established between NF-ĸB activation and the severity of renal disease [10]. In addition, the expression of Bcl2 is majorly detected in the proximal and distal tubules and in the capsular parietal cells in normal rats and humans [17]. In this study Bcl2 expression was significantly reduced in the kidney parenchyma of diabetic control rats, thereby aiding apoptosis. Figure 10 illustrates the suggested mechanisms and pathways that may be involved in the ameliorative effect of AD against kidney damage.

## 4. Materials and Methods

### 4.1. Plant Collection, Authentication, and Preparation

The leaves of AD *Anchomanes difformis* were harvested from a farm in Abeokuta, Nigeria and authenticated at the Herbarium by O.O. Oyebanji; Department of botany and microbiology, University of Lagos, Nigeria, and a specimen was deposited (LUH6623) in the Herbarium. The plant’s name was verified with http://www.theplantlist.org (accessed on 25 March 2020) with the ID: kew-8734. The harvested leaves were dried under shade, milled and de-oiled using n-hexane (10% *w*/*v*) for 2 days. The aqueous extraction was carried out using cold-stirred extraction method, where the defatted leaves were soaked in water (5% *w*/*v*) for 2 days at 2–8 °C. The extract was freeze-dried and stored at −20 °C for further analysis.

### 4.2. Ethical Consideration

Ethical approval for this study was granted by the Research Ethics Committee (REC) of the Faculty of Health and Wellness Sciences, Cape Peninsula University of Technology, Bellville, South Africa (CPUT/HW-REC 2016/A4) and the Ethics Committee for Research on Animals at the South African Medical Research Council (SAMRC), South Africa (REF.04/17), where the animal experiment was conducted.

### 4.3. Animals

Briefly, 64 male Wistar rats weighing approximately 180 ± 10 g were procured for this study from the Animal facility in Stellenbosch University, South Africa. The animals were accommodated at the Primate Unit & Delft Animal Centre (PUDAC), SAMRC and made to adapt to the environment for 3–4 weeks. Housing conditions were controlled: humidity, 45% to 55%; temperature, 22 °C to 26 °C. They were exposed to normal photo period (12 h dark/12 h light) and fed with standard rat chow (SRC). Animal handling, care and other procedures were done in accordance with the standard operating procedure of SAMRC PUDAC (SOP No: 2016-R01) which conforms to the revised South African National Standard for the Care and Use of Animals for Scientific Purposes (South African Bureau of Standards, SANS 10386, 2008).

### 4.4. Modelling and Grouping

**Figure 11:**
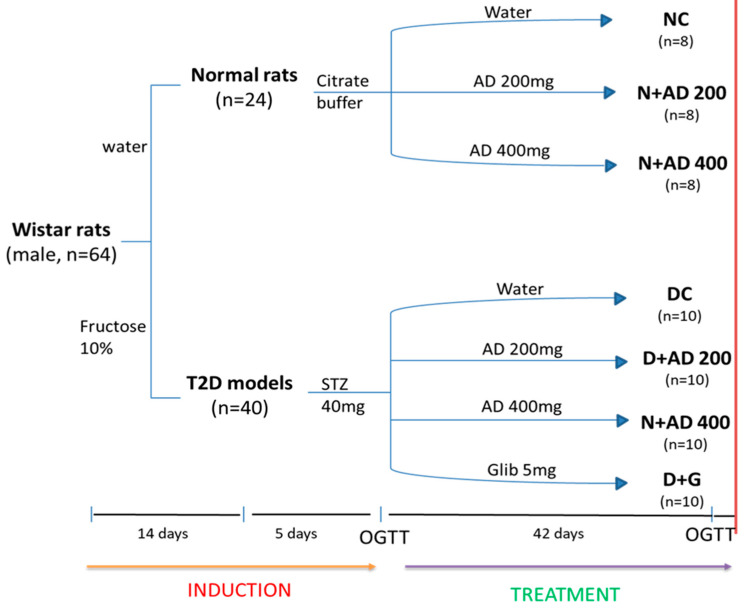
Experimental design. Animals were randomly assigned into 7 groups (n ≥ 8). 14 days administration of 10% fructose preceded a single-dose injection of STZ (40 mg/kg). After 5 days, animals with fasting blood glucose of 15 mmol/L or greater were considered diabetic. OGTT was conducted to confirm insulin resistance. Normal rats were administered the vehicle; water and citrate buffer (CB) correspondingly. Treatment commenced immediately for 42 days via oral gavage. Animals were euthanized on the 43rd day (red bar).

After acclimatization, the rats (with weights between 270 g and 300 g) were indiscriminately grouped into 7 with a minimum of eight rats in each group (Figure 11). The diabetic models received 10% fructose for 2 weeks followed by intraperitoneal injection of 40 mg/KgBW streptozotocin (STZ). Water and citrate buffer served as vehicles for fructose and STZ in normal rats. Group 1 served as the negative controls (non-diabetics) and received vehicle only (NC). Animals in group 2 and 3 served as treatment controls (non-diabetic treated rats) and were placed on 200 mg and 400 mg/KgBW of AD (N+AD 200 and N+AD 400 respectively). Group 4 was the positive control (untreated diabetic rats) and were given vehicle only (DC). Group 5, 6, and 7 were treated diabetic groups placed on 200 mg, 400 mg of AD and 5 mg glibenclamide, respectively (D+AD 200, D+AD 400 and D+G).

### 4.5. Sample Collection

The rats were euthanised after the treatment period. The rats were anaesthetized prior to euthanasia with 2% isoflurane per oxygen (1 L/min flow rate) via inhalation. Blood was collected from the abdominal vein into the Z-serum clot activator tubes. The kidneys and pancreas were immediately harvested, washed in ice-cold phosphate buffer, and weighed. Blood samples were centrifuged at 4000 g for 10 min at 4 °C for serum yield and aliquoted. The serum and tissues were frozen in liquid nitrogen and later stored at −80 °C for further analysis.

### 4.6. Tissue Preparation

The kidney and pancreas samples for histological examination were immediately fixed in 10% buffered formalin solution. Kidney samples that will be used for immunofluorescence assays were fixed with a freezing media, froze in the liquid nitrogen and stored at −80 °C. Homogenization of kidney tissues (200 mg) for other assays were carried out on ice in 2 mls of 50 mM phosphate buffer with 0.5% triton and centrifuged at 10,000× *g* for 15 min at 4 °C. The supernatants were aliquoted and stored at −80 °C.

### 4.7. Estimation of Organ Function and Toxicity Markers

Serum levels of urea and creatinine were measured in the normal and diabetic rats as indices of organ function. The estimation of urea and creatinine in the serum was done on an ABX Pentra 400 Chemistry Analyzer (Horiba) using Horiba kits (Montpellier, France) and performed following the manufacturer’s guidelines. The body weight measurements of all the animals were recorded every week till euthanasia. Relative kidney or pancreas weight for each rat was calculated using the kidney weight or pancreas weight and the weight of the same animal.

$$\mathrm{Relative}\;\mathrm{kidney}/\mathrm{pancreas}\;\mathrm{weight}\;=\;\frac{\mathrm{Kidney}/\mathrm{pancreas}\;\mathrm{weight}\;\left(g\right)}{\mathrm{Total}\;\mathrm{body}\;\mathrm{weight}\;\left(g\right)}\;\times 100\;\%$$

### 4.8. Evaluation of Antioxidant Status and Oxidative Stress Markers

The activities of antioxidant enzymes; CAT and SOD was done in the kidney homogenates according to the method of Ellerby and Bredesen [59]. Other non-enzymic antioxidant indices such as ORAC and FRAP were used to also evaluate the antioxidant status in the kidney. ORAC assay was carried out following the method of Prior and colleagues [60], while FRAP was determined using the method described by Benzie and Strain [61]. Lipid peroxidation was measured to assess oxidative stress status in the kidney by determining TBARS levels in the kidney homogenates using the combined methods of Matsunami et al. and Wasowicz et al. [62,63].

### 4.9. Measurement of Inflammatory Markers

The determination of the levels of interleukins (IL)-1β, IL-6, IL-10, IL-18 and TNFα were determined in the kidney lysate of normal and diabetic rats. Bioplex Promagnetic bead-based assays (Bio-Rad Laboratories, Hercules, USA) were used for the measurement of these inflammatory markers. Assays were performed according to the manufacturer’s instructions (BioRad and Merck Millipore). Bead acquisition and analysis of median fluorescent intensities was done using Bio-Plex Manager software (version 6.0, Hercules, CA, USA).

### 4.10. Quantification of the Expression of Transcription and Apoptotic Proteins

Immunofluorescence staining and imaging was done in the kidney tissues to estimate the expression of transcription factors (Nrf2 and NF-ĸB) and apoptotic proteins (Caspase-3 and Bcl2). Frozen tissues were sectioned (10 µm) on a cryostat (Leica CM 1860 UV Cryostat) and permeabilized with 1× PBS containing 0.025% Triton X-100 (PBS-T). Blocking of tissues was done using 10% normal goat serum in PBS and 5% bovine serum albumin for 2 h. Tissues were incubated with primary antibodies overnight at 4 °C and secondary antibodies for 1 h in the dark. Washes were done after each incubation with PBS-T and PBS, respectively. The slides were mounted (Dako mounting medium, Agilent Technology Inc.,Santa Clara, CA) and imaged with a Zeiss LSM780 ELYRA PS1 super-resolution, confocal microscope with a 10×/0.3 M27 objective (EC “Plan-Neofluar”). The argon multiline laser at 488 nm and DPSS 561-10 laser at 561 nm was used to excite the Alexa Fluor 488 (green) and Alexa Fluor 594 (red) respectively. Zen 2.6 imaging software (blue and black edition, Zeiss Germany) were used for image analysis and to obtain mean fluorescent intensities (MFI) on four images acquired in each experimental condition of three biological repeats.

### 4.11. Histological Examination of the Kidney and Pancreas

The fixed tissues were dehydrated using graded series of alcohol, embedded in paraffin wax, sectioned (5 µm), deparaffinized, rehydrated, and stained with haematoxylin and eosin (H&E) dyes. Slides were mounted and examined under a light microscope. Photomicrographs were taken using digital camera (Motic software, MoticEurope, Barcelona, Spain).

### 4.12. Statistical Analysis

Values are expressed as mean ± standard error of mean (SEM). Statistical analysis of the results was performed using one-way analysis of variance (ANOVA) to find differences between groups. Bonferroni test was used for all pair-wise comparisons. Differences (*F* values) were considered statistically significant at *p* values less than 0.05. All statistical calculations were done using GraphPad Prism Version 5.00 for Windows, GraphPad Inc., San Diego, CA, USA.

## 5. Conclusions

AD enhanced antioxidant status, reduced apoptosis, and modulated inflammatory response in diabetic nephropathy. These biological activities of AD in the kidney might be a useful tool in the prevention and management of diabetic nephropathy and other associated diabetic complications.

## Data Availability

Not applicable.
